# Foresight in clinical proteomics: current status, ethical considerations, and future perspectives

**DOI:** 10.12688/openreseurope.15810.1

**Published:** 2023-04-18

**Authors:** Filip Mundt, Nicolai J. Wewer Albrechtsen, Sebastian Porsdam Mann, Peter Treit, Medini Ghodgaonkar-Steger, Martina O’Flaherty, Reinout Raijmakers, Juan Antonio Vizcaíno, Albert J.R. Heck, Matthias Mann

**Affiliations:** 1 Novo Nordisk Foundation Center for Protein Research, Faculty of Health and Medical Sciences, University of Copenhagen, Copenhagen, Denmark; 2 Department of Clinical Biochemistry, University Hospital, Bispebjerg Hospital, Bispebjerg, Denmark; 3 Faculty of Law, University of Oxford, Oxford, England, UK; 4 Max Planck Institute of Biochemistry, Proteomics and Signal Transduction, Martinsried, Germany; 5 Biomolecular Mass Spectrometry and Proteomics, Bijvoet Centre for Biomolecular Research and Utrecht Institute for Pharmaceutical Sciences, Utrecht University, Padualaan 8, Utrecht 3584 CH, The Netherlands; 6 European Molecular Biology Laboratory, European Bioinformatics Institute (EMBL-EBI), Wellcome Trust Genome Campus, Hinxton, Cambridge, CB10 1SD, UK

**Keywords:** Clinical proteomics; clinical research; ethical challenges

## Abstract

With the advent of robust and high-throughput mass spectrometric technologies and bioinformatics tools to analyze large data sets, proteomics has penetrated broadly into basic and translational life sciences research. More than 95% of FDA-approved drugs currently target proteins, and most diagnostic tests are protein-based. The introduction of proteomics to the clinic, for instance to guide patient stratification and treatment, is already ongoing. Importantly, ethical challenges come with this success, which must also be adequately addressed by the proteomics and medical communities. Consortium members of the H2020 European Union-funded proteomics initiative: European Proteomics Infrastructure Consortium-providing access (EPIC-XS) met at the Core Technologies for Life Sciences (CTLS) conference to discuss the emerging role and implementation of proteomics in the clinic. The discussion, involving leaders in the field, focused on the current status, related challenges, and future efforts required to make proteomics a more mainstream technology for translational and clinical research. Here we report on that discussion and provide an expert update concerning the feasibility of clinical proteomics, the ethical implications of generating and analyzing large-scale proteomics clinical data, and recommendations to ensure both ethical and effective implementation in real-world applications.

## Introduction

Proteins inherently reflect a more functional or phenotypic view of a (liquid) tissue’s status compared to the transcriptome or genome ^1^. Clinics routinely use protein-related information for diagnostic and therapeutic decisions as more than 95% of FDA-approved drugs currently target proteins ^2^ and hundreds of FDA-approved protein-based clinical biomarkers are readily detectable in blood alone ^3^. The stunning development of robust and high-throughput proteomics techniques and workflows in the last decade has broadened access to proteomics, and in conjunction with ever-advancing bioinformatics tools, the biological and translational research fields are increasingly embracing the proteome. Whether a proteome is analyzed by itself or in conjunction with paired nucleic acid ( *proteogenomics*), more proteomes than ever are now being generated. Such readily and economically generated proteomes raise hopes for universal wellness or disease tests that could change the practice of medicine. However, along with promises come potential pitfalls. As a community, we have a responsibility to discuss and address ethical considerations in implementing mass spectrometry (MS)-based proteomics in the clinic.

In the present manuscript, we aim to summarize the fruitful discussions that started at the online Core Technologies for Life Conference (CTLS; https://ctls-org.eu/) conference in September 2021, with a focus on clinical implementation of MS-based proteomics. We also discuss ethical implications and important considerations in acquiring proteomics data from patients, including ‘incidental findings,’ as well as the possibility of re-identifying (anonymized) patient samples. We conclude by providing recommendations for further discussion of the issues raised herein.

**European Proteomics Infrastructure Consortium - Providing Access**

This manuscript is part of the efforts of European Proteomics Infrastructure Consortium - Providing Access (EPIC-XS), which is a project funded by the European Union to provide proteomics expertise and mass spectrometry technology to researchers within the life science arena ( https://epic-xs.eu/). It brings together eighteen institutes, spread across fifteen European countries, with the objective of providing more than 2,400 days of access to high-end proteomics technologies. The EPIC-XS consortium tackles fundamental aspects of the consolidation and enhancement of proteomics technologies and skills across Europe. The EPIC-XS infrastructure contributes to strengthening Europe’s leading role in proteomics by ensuring access of a broad range of researchers to prominent European proteomics facilities, on the premise that research in this field can yield information spanning the whole life science domain, including developments towards potential disease biomarkers. EPIC-XS has an independently appointed ethics advisor to help the consortium ensure the compliance of the project with ethical standards (national and EU) and guidelines of the H2020 program.

## Proteomics in the clinic

A plurality of clinical diagnostics utilize protein-based biomarkers and at large hospitals, there may be as many as three to five million analyses of protein-based biomarkers per year ^3^. However, *proteomics* is still not used in the diagnostic units or in laboratory medicine, for reasons that include perceived technological immaturity, a feared lack of robust and accurate measurements, as well as an apparent high cost. These, in turn, mirror problems seen in other areas of omics research applied to translational medicine, such as a lack of external validation cohorts, insufficient statistical power, and the use of inadequate comparator groups.

In addition, there are additional perceived issues that are specific to clinical proteomic studies. Whereas traditional biomarker-based studies aim to use material from well-controlled standardized clinical trials and from standard operating procedures (SOP), clinical proteomics must be able to handle conditions where the quality of biomaterial sampling and pre-analytical requirements are variable, and sampling procedures are often optimized for measuring devices other than a mass spectrometer. Optimized SOPs may rarely be used for collecting patient material in these settings and therefore, several developments must occur before MS-based proteomics can be successfully implemented in clinical diagnostics. In laboratory medicine, the goal is to provide results that can guide all aspects of patient care, which require robust and reproducible data, even for serial patient samples taken at only a few minutes’ intervals. Several published studies report potential biomarkers of multiple diseases, yet very few, if any, reach clinical application (although proteomics is not alone in this) ^4^. For clinical studies, large sample collections and cohorts are needed, ideally taken by longitudinal sampling. There are several promising developments in this regard, including newer, faster, MS hardware that allows for the analysis of larger cohorts (in the thousands), statistical tools that help to evaluate variance and improve power calculations, as well as meta-analyses of public proteomics data sets ^5– 10^. These developments are complemented by ongoing efforts to increase hardware robustness for large cohorts ^11^, and by targeted MS panels that can be used with relative ease on hundreds, or even thousands of samples for validation efforts ^12^.

Clinical proteomics is likely to play a significant role in clinics by providing a more specific, quantitatively accurate and possibly cheaper platform for diagnostics, as compared to some current gold standards. For example, diagnoses of multiple myeloma or hemoglobinopathies are currently based on gel electrophoresis and liquid chromatography ^13^, which can take up to two weeks. In our experience, MS-based proteomics can do this in hours and, unlike visual detection, is compatible with machine learning approaches, potentially reducing (human) error. In addition, proteomics may directly provide answers that are normally resolved through subsequent genetic testing, for example for hemoglobinopathies 

The potential and difficulties of clinical applications of proteomics are well illustrated by our experience at two major Danish hospitals (Rigshospitalet and Bispebjerg Hospital). Since 2020, we have established an MS-based proteomics setup within the hospital, which includes an EvoSep One liquid chromatography (EvoSep), and an Orbitrap Exploris 480™ mass spectrometer (Thermo Fisher Scientific). In many cases, we have found that MS-based proteomics readily outperforms current clinical diagnostic praxis as a diagnostic tool. Examples include the aforementioned diagnostics of multiple myeloma and hemoglobinopathies, as well as the ability to discriminate between bacterial, viral, and trauma-induced host-related responses. For instance, a simple blood sample can be used to identify patients at high risk of the tick-borne disease borreliosis, as opposed to risky and invasive spinal tap procedure. While it is uncertain how these efforts will be integrated into broad routine clinical practice, physicians in the capital region of Denmark are currently able to request plasma proteomics for routine diagnostic analysis. We are limited by a lack of standardization protocols, or of validation reports in accordance with the standards set forth by the national accreditation institutions as well as by a lack of a universally agreed-upon human proteome standard or reference intervals. Although these issues challenge contemporary MS-based clinical applications, our experience suggests that they are manageable.

Mass spectrometry-based proteomics has created new insights in disease understanding. Landscaping studies have connected somatic mutations to breast cancer signaling ^14^, highlighted subsets of tumors for immune therapy ^15– 17^, identified ALK-fusion diagnostic markers and targets in lung cancer ^15^, and stratified new patient subgroups of medulloblastoma that are invisible via the study of the genome and transcriptome ^18^. Proteomics additionally enables systematic characterization of the extracellular matrix environment ^19^, which is difficult with other omics technologies. In many of these advancements, the enrichment and quantification of post-translational modifications are key, which is again a unique area of study for proteomics.

As a demonstration of the current robustness and reproducibility of quantitative proteomics, three different laboratories (two of which are a partner in EPIC-XS), using different patient cohorts and different methods, independently defined very similar serum proteome signatures that predict disease severity and mortality in COVID-19 patients ^20– 22^. There is considerable work ongoing aiming to standardize these workflows to uphold the International Organization for Standardization (ISO) standard for quality of medical devices (ISO 13485 ^20, 23^). Also in the plasma proteome, post-translational modifications are of fundamental importance, as exemplified by the use of glycoproteins in plasma to improve prediction of patient outcomes ^24^.

In another example, the EPIC-XS center in Copenhagen analyzed plasma samples from nearly 600 patients with alcohol-related liver disease. We were able to predict future liver-related events and all-cause mortality with a Harrell's C-index of 0.90 and 0.79, respectively. Importantly, the diagnostic model performance was reproduced in an independent validation cohort reproduced ^10^, laying the foundation for routine MS-based liver disease testing. Additionally, in a first-of-its-kind publication, we also described a rapid proteomics workflow to analyze and predict new treatment modalities for a patient with terminal cancer, with the outcome approved by the cancer board ^25^. Such analyses might well become standard in future settings of precision oncology and medicine ^26^. We hope to see an era of improved diagnostics, and crucially, improved patient care facilitated by increased use of MS-based proteomics in the clinic.

## Clinical proteomics in the era of the EU General Data Protection Regulation

The General Data Protection Regulation (GDPR) is an EU law governing data protection and privacy. The GDPR is generally applicable and thus also binding for all research carried out partially or wholly in the European Union and the European Economic Area and has served as an inspiration for data privacy legislation globally. It is therefore also relevant for clinical proteomics. Though proteomics information is intended to aid doctors in their decision-making processes, from diagnostics to prognostics and treatment, it can potentially be put to other uses, including the re-identification of individuals in a proteomic dataset. Re-identifiability may be achievable from different data types that can be generated in proteomics experiments, such as protein expression patterns, specific patterns in protein post-translational modifications, and from allotype-induced amino-acid sequence variation (including single amino acid variants) ^27^. Presently, sequence information from proteomics data is less likely to re-identify an individual than genomic or transcriptomic sequence data ^28, 29^. The re-identification of an individual may enable the derivation of sensitive information about that individual or other infringements of privacy. More pertinently, such data typically contains several incidental findings; the question of whether to return these findings hinges on the medical actionability of the finding ( *e.g.,* biomarkers), as well as patient preference. This is true even for retrospective proteomics studies ( *i.e.*, reanalysis of past data, which is available in the public domain).

Identifiable information is information, such as telephone or social security numbers, birthdates, and addresses that might be used to identify a unique individual. By contrast, re-identifiable information is information that enables one to match one sample to another in a dataset. Re-identifiable information has the potential to become identifiable when combined with metadata that is itself identifiable. In one such effort to reanalyze proteomics data from a previously published longitudinal weight loss study of 1,500 individuals over 14 months, we found plasma proteomes uniquely re-identifiable within the cohort based on individual-specific protein expression levels as well as allotype-specific variant peptides ^21, 30^. Additionally, vitamin D-binding proteins vary significantly based on ethnic background and can therefore be used to derive the ethnicity of study participants, which is also readily apparent from the distribution of coding single nucleotide variants (cSNPs) ^31^. Levels of pregnancy-zone protein could be used to determine whether an individual is pregnant or post-menopausal, and various hormone-related proteins can also be used to identify biological sex. These findings illustrate the potential for deriving sensitive information about individuals from their proteomes, provided sufficient metadata is available. These conclusions are ethically significant since such information could in theory be used to disadvantage or discriminate against individuals.

In the same weight loss study, the APOE allotype status could be determined from the proteomic data. APOE alleles are associated with different cardiovascular and Alzheimer’s disease risks ^30^. The APOE4 allele strongly increases the risk of Alzheimer’s disease, whereas the APOE2 allele is associated with increased cholesterol levels and cardiovascular pathologies. Similarly, the presence of elevated levels of glycated forms of hemoglobin protein can indicate (pre-) diabetic pathology. Likewise, the levels of proteins associated with inflammation, lipid metabolism, and various hormones, could all point to different likely health and disease states, which is indeed the primary goal of plasma proteome profiling. Complicating the question of what should be done with such information is the fact that only some of these (e.g., glycated hemoglobin and APOE2 in the examples above) are actionable, meaning that this knowledge could benefit patient’s health and wellbeing, whereas some ( *e.g*., APOE4) are currently unactionable and may instead lead to distress, when this information is communicated to the patient; actionable information is information that, if returned to individuals, can be acted on to their benefit.

In the Utrecht EPIC-XS site, alternative plasma proteomics approaches are being explored to monitor immunoglobulin repertoires ^32^ and proteoform profiles ( *i.e*., the full compendium of different forms of a protein, including protein species that contain different sets of post-translational modifications) or serum glycoproteins ^24^. In these studies, small liquid biopsies (blood, human milk) are taken from individual patients, whereafter either specific classes of immunoglobulins or selected plasma glycoproteins are extracted, which are subsequently analyzed and identified by MS. These preliminary studies revealed that such traits of the plasma proteome can be rather unique for each donor and thus potentially even more tractable than data obtained by protein expression profiling described above. These traits are not directly traceable to the genome sequences of the donor and thus represent more unique features of the donor’s proteome. It needs to be seen whether such data also provides actionable results. However, incidentally, our analyses have already led to the discovery of a B-cell malignancy in one of the donors, which led the doctors to change therapy. Although these approaches were performed using ‘top-down proteomics’, a still less mature technology than the more standard ‘bottom-up’ expression proteomics pipelines described above, they may point to additional issues that need to be addressed from an ethical angle.

Even with these risks, sharing clinical proteomics data, with consent from the patients, is the goal for most proteomics researchers. There are already standardized community guidelines for data submission and dissemination of proteomics data in the public domain ^33, 34^, which were developed under the umbrella of the ProteomeXchange Consortium, and, are tied to the requirements from either funding agencies, scientific journals - or both. The current community standard is to upload MS raw files and the processed results to the PRoteomics IDEntifications database (PRIDE, at the European Bioinformatics Institute [EMBL-EBI]), which is the leading resource within ProteomeXchange ^33, 34^. The wide availability of public data also tends to increase the quality of data and clinical research because of more transparent procedures and possibilities for proteomics data re-use including meta-analyses, among many other applications [28118949]. The overall goal is to make the data findable, accessible, interoperable, and reusable after the (FAIR) principles ^35^.

In the EU, the GDPR has strengthened privacy protection but, as a consequence this has made more difficult to enable sharing of data between academic research institutes, which was not necessarily the original intention in the medical context. The GDPR regulations are complemented by other regional or national regulations such as the Health Insurance Portability and Accountability Act (HIPAA) in the United States of America, and the Act on the Protection of Personal Information (APPI) in Japan. The current state of the art with respect to data management practices of sensitive human data in proteomics has been summarized recently ^28^.

To illustrate the unintended consequences of the GDPR, we have experienced a direct example of a clash between the proteomics community guidelines and GDPR as part of a large national collaboration in Denmark where we analyzed plasma samples from patients with alcohol-related liver disease ^10^. In this study, we planned to follow the ProteomeXchange guidelines, by uploading the MS raw files, as well as standard clinical data ( *e.g*., age and body–mass index), to the PRIDE database. However, the hospital's compliance officers concluded that this data qualified as personal clinical data, the publication of which required specific permission that that not available at the time. After considerable delay (almost a year), we resolved the issue by providing the data in a “controlled access” manner, meaning that a user wanting to get access to the data has to apply for access at the Danish Data Protection Agency (DDPA) and be approved by an ethics committee for the Region of Southern Denmark, which is a lengthy process. ProteomeXchange resources including PRIDE were originally set up to contain fully open data (similarly to resources such as GEO ^36^ and ArrayExpress ^37^ in the transcriptomics field). PRIDE does not currently offer controlled access. Other resources such as the European Genome-phenome Archive (EGA) do, but they were set up for DNA/RNA sequencing data and their data model is tailored for this functionality. PRIDE has started to work in a controlled access repository for proteomics data. This will still take some time to be in production, but it is hoped to help researchers to solve the issues that they are facing in this context, and at the same type providing tailored support for proteomics data. Obtaining controlled access data is a generally lengthy and cumbersome process—not an issue exclusive to proteomics. This will unfortunately hinder many researchers from accessing and ultimately using the data and is thus arguably itself of ethical concern. It might also lead to a bias in who will use it, with smaller institutions lacking funding and resources to acquire access, thus favoring large, well-funded institutions.

## How is ethical analysis relevant to a forthcoming era of clinical proteomics?

We recently surveyed the recent discussion of ethics in clinical proteomics and identified four reasons to engage seriously with the topic ^38^. Among the main findings were, first, that ethics is also about finding ways to increase the beneficial impact of the work that we do, rather than only an obstacle to be overcome. Second, experience from related fields, especially genomics, shows that early discussion of these issues can be beneficial, because informed self-regulation has numerous advantages over the alternative of having external legislation being imposed from those not within the field of proteomics. Third, we can learn from the way similar issues have been handled in genomics and other fields; and fourth, despite this similarity, it is important to consider as early as possible which issues might be different and unique to the clinical proteomics context, when compared with nucleotide sequencing data.

The discipline of bioethics provides a framework useful for identifying and discussing ethical issues emerging by innovations in biotechnology and medicine. The bioethical methodology involves the application of normative principles gleaned from ethical traditions to scientific and medical contexts. These principles, briefly, are: (i) beneficence, which stresses the ethical value of benefiting people; (ii) non-maleficence, which conversely concerns the ethical imperative of not harming persons; (iii) justice, or the value of treating people fairly and equitably; and (iv) autonomy, requiring respect for the free and informed decisions of individuals. Although these principles do not themselves provide a method for determining what ought to be done, they do provide a framework useful for clarifying underlying issues of ethical importance.

Using these bioethical principles as operational definitions of ‘ethical issues’ (an issue is ethical if it is identified as such or obviously relates to these principles) we performed a systematic review of ethical issues already discussed in the clinical proteomics literature. By employing qualitative analysis, we found 40 ethical issues across 16 included studies and grouped these into 10 ethical themes, varying from the importance of standards and quality control to the need for international discussion and development of guidelines on ethical issues in clinical proteomics ^38^. The themes also included incidental findings, re-identifiability, and the potential for discovery of sensitive information previously identified as ethically important topics, as described above ^39^.

Most of these issues had already been discussed in the context of clinical genomics. As a result, there is a high degree of consensus on important topics in this field. The most pertinent lessons from genomics concern the handling of sensitive data and incidental findings, as well as the importance of early and serious discussion of ethical issues to avert subsequent externally imposed regulations. For example, the distinction between actionable and unactionable information is of great ethical and practical significance. Reviews in the literature generally concluded that actionable information ought to be returned to the individual or their health care provider, whereas unactionable information should not ^40, 41^. These reviews also argued that individuals should be informed of the likelihood of such findings at the point of consent and that individuals' preferences as expressed during the consent process regarding the return of findings should be respected. The literature on the return of incidental findings in genomics also pointed out that attempts at determining the actionability and health relevance of findings are hampered by the high prevalence of variants of uncertain significance. There are additional issues concerning whether incidental findings should be routinely incorporated in health registries and other scientific databases as well as what should be done with incidental findings arising from the reanalysis of old data. Most of these topics will be relevant for proteomics and it is clear in our view that the field can certainly learn a great deal from that discussion.

One area of divergence between genomics and proteomics is the ability of the latter to capture phenotypic information. Proteomic information can used to provide actionable health information to individuals and to advance biomedical understanding. Notably, this can be done whatever the health state, and healthy individuals could also receive health-relevant information, which they could incorporate into their daily lifestyles if they wished to do so. However, these profiles should be treated with caution as they could contain all kinds of potentially sensitive information, much of which will be unrelated to the reasons why people chose to be profiled. What, then, should be done with this extra information? A simple method of avoiding these ethical questions is simply not to look for incidental findings or to anonymize data sets completely. However, failing to analyze or share health-related information can result in missed opportunities to improve the individual’s and other people's lives.

Serious, sustained, and early treatment of these and other ethical issues are likely to yield sustained benefits by informing the development of guidelines on ethical issues by the clinical proteomics community. Self-regulation in clinical proteomics is not only important because it directly involves the scientists' expertise in the field but also because guidelines developed through internal consensus are more likely to be perceived as legitimate and therefore be effective.

## Conclusion

The purpose of the clinical proteomics round table discussion that constitutes the foundation of this paper was to start a comprehensive conversation aimed at identifying key challenges and proposing solutions to maintain the trustworthiness and effectiveness of clinical proteomics. Efforts towards facilitating these goals will help serve in incorporating clinical proteomics technologies in all aspects of patient management. Though many critical questions were raised during our discussion, and while it was clear to all that there is a long and potentially difficult road ahead, there was nevertheless broad consensus on the bright outlook for clinical proteomics as a strong asset to clinicians. By communicating the benefits, clarifying SOPs, and initiating discussions at international scientific meetings, we hope to discuss and eventually resolve some of the ethical and regulatory issues that we are currently hampered by. In particular, we propose:

- A natural follow-up of EPIC-XS, *i.e*., a strong joint EU proteomics infrastructure community that should work toward setting up SOPs for MS-based clinical proteomics workflows and methods, in accordance with the standards set forth by the national medical accreditation institutions in various EU countries. The aim should be not only to increase throughput and reduce costs but also to work according to the FAIR principles whenever possible given the GDPR guidelines and to generate robust and reproducible data across different centers.
- Addressing the need to develop tailored bioinformatics infrastructure to handle and manage sensitive human (clinical) proteomics data. The most urgent development is the availability of controlled access functionality in public proteomics data repositories. Controlled access functionality is already available for DNA/RNA sequencing data in resources such as the EGA, but their data model was originally developed for DNA/RNA sequencing data and cannot appropriately represent proteomics datasets. Although the first step would be the availability of a central controlled access data infrastructure, there will be a need to build on existing infrastructures that are amenable to cross-border data sharing without the data actually crossing borders e.g. the European Life-sciences Infrastructure for biological Information (ELIXIR) ( https://elixir-europe.org/) national nodes ( *e.g*. a federated infrastructure). This could be achieved under an umbrella structure and involve partners such as EMBL-EBI, the Biobanking and BioMolecular resources Research Infrastructure-European Research Infrastructure Consortium (BBMRI-ERIC), or European Strategy Forum on Research Infrastructures (ESFRI), and/or ELIXIR, among others.
- Data access committees are key since they provide access to researchers for human-sensitive datasets in the public domain. We need to strengthen our dialogues with clinicians, patient interest groups, and data access committees about the particularities of proteomics data which may generally be less risky for patient re-identification, when compared with nucleotide sequencing information. There is also a need for further research studies in this area to provide as much accurate and useful information as possible to take properly informed decisions.
- Developing clear guidelines on how to handle human-sensitive clinical proteomics data. Importantly, they should address how to handle “incidental findings”, both actionable and unactionable, when searching or re-searching proteomics data. Furthermore, guidelines will need to be developed concerning the submission and dissemination of such data across public resources. This needs to be done in collaboration between clinicians, patient interest groups, bioethicists, data protection specialists, and proteomics experts.

These are all mid- to long-term objectives on which our sibling platforms in genomics and transcriptomics have been working for years. From our discussions, it also became clear that there needs to be a more concerted effort not only in Europe but ideally worldwide in formulating a strategy wherein clinical proteomics can achieve sustainability. These and other issues could be addressed at one of the large annual conferences attended by a significant portion of the community, such as the Human Proteome Organization (HUPO) world congress.

Proactively addressing the above-mentioned challenges in a cohesive way would lead to the development of valuable and appropriate guidelines for the clinical proteomics community. This would be preferable to having external governance impose restrictions on the field. We believe that this is a very important goal to maximize the benefit of the coming revolution in clinical proteomics for the individual and for society.

## Data Availability

No data are associated with this article.
