# Supplementary material for: Foresight in clinical proteomics: current status, ethical considerations, and future perspectives
Source: Open Res Eur. 2023 Nov 1;3:59. Originally published 2023 Apr 18. [Version 2] doi: 10.12688/openreseurope.15810.2 (PMC10446044; doi:10.12688/openreseurope.15810.2)
Supplement: Peer review history

# Reviewer report: Reviewer response for version 2

**DOI:** 10.21956/openreseurope.18137.r35993

**Published:** 2023-11-02

**Authors:** Nurhan Ozlu, Daniel J. Geiszler

**Affiliations:** 1 Department of Molecular Biology and Genetics, Koc Universitesi, Istanbul, İstanbul, Turkey; 2 Department of Molecular Biology and Genetics, Research Center for Translational Medicine, Koc Universitesi, Istanbul, İstanbul, Turkey

The revised version addressed all our comments.

Where applicable, are recommendations and next steps explained clearly for others to follow? (Please consider whether others in the research community would be able to implement guidelines or recommendations and/or constructively engage in the debate)

Yes

Does the article adequately reference differing views and opinions?

Yes

Are all factual statements correct, and are statements and arguments made adequately supported by citations?

Partly

Is the rationale for the Open Letter provided in sufficient detail? (Please consider whether existing challenges in the field are outlined clearly and whether the purpose of the letter is explained)

Yes

Is the Open Letter written in accessible language? (Please consider whether all subject-specific terms, concepts and abbreviations are explained)

Yes

Reviewer Expertise:

Proteomics, computational proteomics, cell biology

We confirm that we have read this submission and believe that we have an appropriate level of expertise to confirm that it is of an acceptable scientific standard.

# Reviewer report: Reviewer response for version 1

**DOI:** 10.21956/openreseurope.17080.r34062

**Published:** 2023-08-14

**Authors:** Kellye A Cupp-Sutton

**Affiliations:** 1 The University of Oklahoma, Norman, Oklahoma, USA

This open letter by Mundt *et al.* discusses the various current and potential application of proteomics methods for clinical proteomics. The authors discuss in detail the ethical implications of untargeted and targeted proteomics on anonymized clinical samples. The letter is well written and insightful with in depth analysis of many aspects presented for ethical consideration by the readers. The examples presented for how the ethical considerations have already become an issue in current research serves to further demonstrate the urgency of the discussion. Moreover, the authors present several actionable suggestions for how to move the application of clinical proteomics forward.

 I strongly recommend approval of this manuscript.

Where applicable, are recommendations and next steps explained clearly for others to follow? (Please consider whether others in the research community would be able to implement guidelines or recommendations and/or constructively engage in the debate)

Yes

Does the article adequately reference differing views and opinions?

Yes

Are all factual statements correct, and are statements and arguments made adequately supported by citations?

Yes

Is the rationale for the Open Letter provided in sufficient detail? (Please consider whether existing challenges in the field are outlined clearly and whether the purpose of the letter is explained)

Yes

Is the Open Letter written in accessible language? (Please consider whether all subject-specific terms, concepts and abbreviations are explained)

Yes

Reviewer Expertise:

Top-down proteomics, analytical biochemistry

I confirm that I have read this submission and believe that I have an appropriate level of expertise to confirm that it is of an acceptable scientific standard.

# Reviewer report: Reviewer response for version 1

**DOI:** 10.21956/openreseurope.17080.r34055

**Published:** 2023-08-07

**Authors:** Maria Hernandez-Valladares

**Affiliations:** 1 University of Granada, Granada, Spain

This Open Letter introduces to the reader some of the challenges we are facing in clinical proteomics. Although most of them are mentioned, the authors mainly focused on very important ethical considerations. I should say that I have missed some more discussion and future perspectives on some other crucial points such as how we could overcome the lack of external validation cohorts and exchange samples, and how to improve the use of more proper comparator groups.

 The authors describe some examples on proteomics-based clinical assays and some clashes with the General Data Protection Regulation (GDPR) in Denmark. Some more examples in other countries would have been much appreciated.

Where applicable, are recommendations and next steps explained clearly for others to follow? (Please consider whether others in the research community would be able to implement guidelines or recommendations and/or constructively engage in the debate)

Yes

Does the article adequately reference differing views and opinions?

Yes

Are all factual statements correct, and are statements and arguments made adequately supported by citations?

Yes

Is the rationale for the Open Letter provided in sufficient detail? (Please consider whether existing challenges in the field are outlined clearly and whether the purpose of the letter is explained)

Yes

Is the Open Letter written in accessible language? (Please consider whether all subject-specific terms, concepts and abbreviations are explained)

Yes

Reviewer Expertise:

Clinical proteomics

I confirm that I have read this submission and believe that I have an appropriate level of expertise to confirm that it is of an acceptable scientific standard.

# Response

**Published:** 2023-10-24

**Authors:** Filip Mundt

**Affiliations:** Faculty of Health and Medical Sciences, University, Novo Nordisk Foundation Center for Protein Research, Copenhagen, Denmark

**We wish to express our gratitude to the reviewers for their thoughtful and constructive feedback, which has improved the quality of this manuscript. We welcome their input and in the sections that follow, we aim to address each of their comments and suggestions, reflected in the updated manuscript.****

*1. This Open Letter introduces to the reader some of the challenges we are facing in clinical proteomics. Although most of them are mentioned, the authors mainly focused on very important ethical considerations. I should say that I have missed some more discussion and future perspectives on some other crucial points such as how we could overcome the lack of external validation cohorts and exchange samples, and how to improve the use of more proper comparator groups.*

**This is indeed a valid point, and in an ideal scenario, every clinical proteomics study should incorporate an external validation cohort. While there have been instances where parallel efforts have corroborated ongoing clinical proteomics studies (as exemplified by Archer et al., 2018, and Forget et al., 2018), the prevailing publishing environment can present challenges in this regard. Additionally, the successful exchange of reference samples between proteomic laboratories, as demonstrated by Mertins et al. in 2018, has been an exception rather than the norm. We have added a brief discussion of this topic, to further address and highlight this issue.**  

*2. The authors describe some examples on proteomics-based clinical assays and some clashes with the General Data Protection Regulation (GDPR) in Denmark. Some more examples in other countries would have been much appreciated.*

**In our collective experience, we have observed varying degrees of stringency in the application of GDPR regulations to omics data across different EU countries. While we do not possess more specific recent examples, we are concerned that such instances may accumulate unless we, as a community, proactively address the challenges outlined in this work.**

# Reviewer report: Reviewer response for version 1

**DOI:** 10.21956/openreseurope.17080.r31191

**Published:** 2023-05-16

**Authors:** Nurhan Ozlu, Daniel J. Geiszler

**Affiliations:** 1 Department of Molecular Biology and Genetics, Koc Universitesi, Istanbul, İstanbul, Turkey; 2 Department of Molecular Biology and Genetics, Research Center for Translational Medicine, Koc Universitesi, Istanbul, İstanbul, Turkey

Mundt *et al.,* provide important insight into the role and possible implementations of proteomic tools in clinics and the ethical aspects of clinical proteomics. The manuscript was written based on a discussion of EPIC-XS members at the Core Technologies for Life Sciences (CTLS) conference.

 The authors provide an extensive discussion about the ethical implications of generating and analyzing large-scale proteomic data in clinics. As pioneers in the field, with multiple studies where they successfully apply proteomics in clinics, the authors provide a timely discussion about how to put proteomics on the right track to be used routinely in clinics with the appropriate ethical policies. The authors pointed out some important concepts as re-identification of individuals in a proteomic data set, where one would think that given the dynamic nature of proteins that may not be an issue however, authors provide important evidence from different studies about this possibility. Development in proteomic and computational technologies will likely improve its capacity in the future. 

 The authors also emphasize the ethical problems surrounding informing the patients about incidental findings which may be useful for the patient’s treatment. They raised many points that are shared with genomics studies while also highlighting some unique issues in proteomics. 

 They also mention an initiation of “controlled access” option for PRIDE for clinical data and its possible impact in the future. This will be interest of several proteomic and clinical researchers. 

 The authors also make proposals for future steps that can be taken to address clinical and ethical issues in proteomics. These include setting up EU-wide SOPs for MS-based clinical proteomics; developing tailored bioinformatics infrastructure for sensitive data; improving communication between clinicians, patient interest groups, and data access committees; and developing clear guidelines for incidental findings and sensitive data.

 We strongly recommend the indexing of this manuscript.

 We suggest the following improvements for the manuscript where it is possible before the indexing:

1. The authors talk about the limitations of proteomic applications in clinics but also point out the potential of clinical proteomics when it is combined with machine learning approaches and the power of artificial intelligence. The authors may elaborate on this further and provide by providing some references. More specifically following sentences require references:
*“In our experience, MS-based proteomics can do this in hours and, unlike visual detection, is compatible with machine learning approaches, potentially reducing (human) error. In addition, proteomics may directly provide answers that are normally resolved through subsequent genetic testing, for example for hemoglobinopathies”*
2. The following paragraph is an introductory to the proteomics which needs to be discussed earlier in the text, before going to specific application and should be given with references:
  “ *Proteomics additionally enables systematic characterization of the extracellular* *matrix environment ^19^, which is difficult with other omics technologies. In many of these advancements, the enrichment and quantification of post-translational modifications are key, which is again a unique area of study for proteomics.”*
3. This reference should be inserted properly, *”[28118949]”*
4. Authors mention the importance of international effort in establishing appropriate platforms for clinical proteomics by developing clear guidelines for ethical issues. Perhaps the importance of multi-national effort should also be emphasized in the following sentence:
  “ *This needs to be done in collaboration between clinicians, patient interest groups, bioethicists, data protection specialists, and proteomics experts.”*
  The countries which do not have much proteomic or computational expertise but rather provide biological samples of some rare or endemic diseases in international collaborations will greatly benefit from ethical guidelines for clinical proteomics that take into account cultural sensitivities.
5. *“Clinics routinely use protein-related information for diagnostic and therapeutic decisions as more than 95% of FDA-approved drugs currently target proteins* *^2^* *and hundreds of FDA-approved protein-based clinical biomarkers are readily detectable in blood alone* *^3^* *.” - *This sentence implies causality (“Clinics routine use protein-related information … AS … drugs currently target proteins”), but biomarkers do not need to be drug targets to be useful. It should be slightly rephrased.
6. *“Core Technologies for Life Conference (CTLS;* *https://ctls-org.eu/* *) conference in September 2021”* - Should this read “Core Technologies for Life Sciences conference”?
7. “ *Several published studies report potential biomarkers of multiple diseases, yet very few, if any, reach clinical application (although proteomics is not alone in this)* *^4^* *.”* - It would be more accurate here to say “Many published studies”.
8. *“ideally taken by longitudinal sampling.”* - Is this true in all cases? Why is it true? A reference here might be clarifying for readers.
9. References 5-10 would be more useful if distributed among the individual items they describe in the sentence, e.g., “statistical tools that help to evaluate variance and prove power calculations(5,6)”.
10. *“In our experience, MS-based proteomics can do this in hours and, unlike visual detection, is compatible with machine learning approaches, potentially reducing (human) error.”* - This sentence is not clear. What is “visual detection”? Machine-learning based image classification approaches for diagnosis are a large and active area of research, e.g., Ker et al., (2019) ^1^ . 
11. The authors mention experience at two major Danish hospitals (Rigshospitalet and Bispebjerg Hospital) but only provide an experience at one hospital, and it is not clear which of the two hospitals is being discussed.
12. “For instance, a simple blood sample can be used to identify patients at high risk of the tick-borne disease borreliosis, as opposed to risky and invasive spinal tap procedure.” - Can a citation be provided?
13. “Presently, sequence information from proteomics data is less likely to re-identify an individual than genomic or transcriptomic sequence data ^28, 29^.” - This statement is true. However, it is important to note that improvements in computational methods may lead to data becoming re-identifiable (or identifiable) in retrospective studies even if it was not originally re-identifiable (or identifiable) when it was made public.
14. “By contrast, re-identifiable information is information that enables one to match one sample to another in a dataset.” - This sentence comes after a paragraph devoted to re-identifying patients based on proteomics data. It would be better if it were placed before the discussion of patient re-identification.

Where applicable, are recommendations and next steps explained clearly for others to follow? (Please consider whether others in the research community would be able to implement guidelines or recommendations and/or constructively engage in the debate)

Yes

Does the article adequately reference differing views and opinions?

Yes

Are all factual statements correct, and are statements and arguments made adequately supported by citations?

Partly

Is the rationale for the Open Letter provided in sufficient detail? (Please consider whether existing challenges in the field are outlined clearly and whether the purpose of the letter is explained)

Yes

Is the Open Letter written in accessible language? (Please consider whether all subject-specific terms, concepts and abbreviations are explained)

Yes

Reviewer Expertise:

Proteomics, computational proteomics, cell biology

We confirm that we have read this submission and believe that we have an appropriate level of expertise to confirm that it is of an acceptable scientific standard.

## References

[1] : Automated brain histology classification using machine learning. *J Clin Neurosci* .2019;66: 10.1016/j.jocn.2019.05.019 239-245 10.1016/j.jocn.2019.05.019 31155342

# Response

**Published:** 2023-10-24

**Authors:** Filip Mundt

**Affiliations:** Faculty of Health and Medical Sciences, University, Novo Nordisk Foundation Center for Protein Research, Copenhagen, Denmark

**We wish to express our gratitude to the reviewers for their thoughtful and constructive feedback, which has improved the quality of this manuscript. We welcome their input and in the sections that follow, we aim to address each of their comments and suggestions, reflected in the updated manuscript.**  

*We suggest the following improvements for the manuscript where it is possible before the indexing:*  

*1. The authors talk about the limitations of proteomic applications in clinics but also point out the potential of clinical proteomics when it is combined with machine learning approaches and the power of artificial intelligence. The authors may elaborate on this further and provide by providing some references. More specifically following sentences require references:* *“In our experience, MS-based proteomics can do this in hours and, unlike visual detection, is compatible with machine learning approaches, potentially reducing (human) error. In addition, proteomics may directly provide answers that are normally resolved through subsequent genetic testing, for example for hemoglobinopathies”*

**The above statement is grounded in our extensive clinical experience with a mass spectrometer for proteomics within this particular clinical context. We have not published on these findings, and are currently unable to provide a reference.**            

 2. The following paragraph is an introductory to the proteomics which needs to be discussed earlier in the text, before going to specific application and should be given with references: *“Proteomics additionally enables systematic characterization of the extracellular matrix environment19, which is difficult with other omics technologies. In many of these advancements, the enrichment and quantification of post-translational modifications are key, which is again a unique area of study for proteomics.”*

**In response to your query, we have relocated and elaborated on this section, placing it at an earlier point in the document.**  

*3. This reference should be inserted properly, ”[28118949]”*

**The reference has been formatted properly.**    

*4. Authors mention the importance of international effort in establishing appropriate platforms for clinical proteomics by developing clear guidelines for ethical issues. Perhaps the importance of multi-national effort should also be emphasized in the following sentence:* *“This needs to be done in collaboration between clinicians, patient interest groups, bioethicists, data protection specialists, and proteomics experts.”*    

**Ethical guidelines for the management of proteomics data will play an increasingly crucial role, and it is imperative to consider cultural sensitivities when adapting these guidelines to various countries and legislations. As outlined, there is no need to start from scratch, as substantial groundwork has already been laid in the context of DNA/RNA sequencing data. We have added on this important issue of the need of multi-national efforts.**  

*5. “Clinics routinely use protein-related information for diagnostic and therapeutic decisions as more than 95% of FDA-approved drugs currently target proteins2 and hundreds of FDA-approved protein-based clinical biomarkers are readily detectable in blood alone3.” - This sentence implies causality (“Clinics routine use protein-related information … AS … drugs currently target proteins”), but biomarkers do not need to be drug targets to be useful. It should be slightly rephrased.*

**We agree with this comment, and the section has been rephrased to address this. **  

*6. “Core Technologies for Life Conference (CTLS; https://ctls-org.eu/) conference in September 2021” - Should this read “Core Technologies for Life Sciences conference”?*

**This has been amended.**  

*7. “Several published studies report potential biomarkers of multiple diseases, yet very few, if any, reach clinical application (although proteomics is not alone in this)4.” - It would be more accurate here to say “Many published studies”.*

**We fully agree, and it has been changed.**  

*8. “ideally taken by longitudinal sampling.” - Is this true in all cases? Why is it true? A reference here might be clarifying for readers.*

**We argue that since diseases exhibit adaptability, progression, and dynamism over time, then capturing disease dynamics would often benefit from longitudinal profiling rather than relying on single-time-point measurements. Longitudinal studies track changes over time, offering a more accurate understanding of disease progression and response to treatments.**      

 9. References 5-10 would be more useful if distributed among the individual items they describe in the sentence, e.g., “statistical tools that help to evaluate variance and prove power calculations(5,6)”.

**Good point, and we have now separated those references.**  

 10. “In our experience, MS-based proteomics can do this in hours and, unlike visual detection, is compatible with machine learning approaches, potentially reducing (human) error.” - This sentence is not clear. What is “visual detection”? Machine-learning based image classification approaches for diagnosis are a large and active area of research, e.g., Ker et al., (2019)1 .

**Still in clinics today, visual evaluation of test results is a reality. Computerised image analyses are obviously moving forward, but have had some issues, and implementation has not been ubiquitous, yet. We have clarified, and added references.****

*11. The authors mention experience at two major Danish hospitals (Rigshospitalet and Bispebjerg Hospital) but only provide an experience at one hospital, and it is not clear which of the two hospitals is being discussed.*

**The proteomics laboratory initiated at the Rigshospitalet, has now moved to Bispebjerg. This has been clarified in the manuscript.**  

*12. “For instance, a simple blood sample can be used to identify patients at high risk of the tick-borne disease borreliosis, as opposed to risky and invasive spinal tap procedure.” - Can a citation be provided?*

**Unfortunately, we cannot provide a reference currently. This is based on ample clinical experience and yet unpublished data from our groups.**  

*13. “Presently, sequence information from proteomics data is less likely to re-identify an individual than genomic or transcriptomic sequence data28,29.” - This statement is true. However, it is important to note that improvements in computational methods may lead to data becoming re-identifiable (or identifiable) in retrospective studies even if it was not originally re-identifiable (or identifiable) when it was made public.*

**Indeed, your point is valid. A paragraph has been included to further spotlight this perspective.**  

 14. “By contrast, re-identifiable information is information that enables one to match one sample to another in a dataset.” - This sentence comes after a paragraph devoted to re-identifying patients based on proteomics data. It would be better if it were placed before the discussion of patient re-identification.

**Good point. We have moved this section now.**
